# Efficacy and positive outcome of physical activity in pregnant women

**DOI:** 10.12669/pjms.38.8.4911

**Published:** 2022

**Authors:** Uchenna Benedine Okafor, Daniel Ter Goon

**Affiliations:** 1 Uchenna Benedine Okafor, Department of Nursing Science, University of Fort Hare, East London, South Africa; 2 Prof. Daniel Ter Goon, Department of Public Health, University of Fort Hare, East London, South Africa

**Keywords:** Physical activity, Self-efficacy, Positive attitude, Pregnant women

## Abstract

**Background & Objective:**

Evidence has shown the innumerable benefits of prenatal physical activity practice; therefore, the knowledge pregnant women have, and efforts to sustain the knowledge and encourage them to engage in prenatal physical activity, are desirable. The objective of the study was to assess the knowledge and attitudes concerning prenatal physical activity of pregnant women attending primary health antenatal care clinics.

**Methods:**

A cross-sectional study of 1082 pregnant in Buffalo City Municipality, Eastern Cape province, South Africa were sampled from July to October 2019. Socio-demographic and maternal characteristics, and knowledge, attitude and practices towards prenatal activity were obtained.

**Results:**

Overall, 62.4% women had high knowledge regarding prenatal physical activity; and half of the women showed a positive attitude toward it (50.1%). The majority of the participants affirmed prenatal physical activity is safe (88.2%) and beneficial for the baby (79.6%). Whilst participants had knowledge of other types of antenatal exercises, 80.9% of the women had no knowledge of swimming exercise. Negative attitudes towards physical activity included the feeling of tiredness (67.7%), lack of interest (64.8%), and inadequate information on physical activity (59.5%).

**Conclusions:**

The women had high knowledge of prenatal physical activity, and relatively positive attitudes toward prenatal physical activity. The feeling of tiredness, lack of motivation and inadequate information on physical activity constituted negative attitudes towards physical activity. There is need to provide education and advocacy in the clinical settings; also, interventions to encourage and promote prenatal physical activity in the community and at home are desirable to address the weaknesses identified in this study regarding the women’s knowledge and attitudes concerning prenatal physical activity.

## INTRODUCTION

Physical activity or exercise can be viewed as an antidote to the health challenges of populations. However, previously, physical activity participation by women was not a widely acceptable norm, as the society expressed disapproval and reservations about women’s involvement in any physical activity; primarily, this was underpinned by cultural and safety concerns. However, the negative perception regarding the safety of prenatal physical activity has improved, or rather, changed: attitudes toward physical activity or exercise in pregnancy have improved significantly. Today, scientific evidence has counteracted the erroneous beliefs of linking prenatal physical activity with maternal adverse health outcomes, such as preterm birth, low birth weight, miscarriage and perinatal mortality.^1^-^4^ In other words, prenatal physical activity practice has minimal or rare adverse health complications,^5^ as previously thought.

Participation in physical activity during pregnancy is beneficial to health as widely reported in the literature. Notably, studies have shown that physically active pregnant women are less likely to have a caesarean section and quick postpartum recovery time,^6^,^7^ and chances of harm to the mother and baby are minimal.^8^ Women who partake of physical activity also find it reduces fatigue, stress, anxiety, and depression,^9^-^11^ low back pain,^12^-^14^ and self-efficacy, and better body image.^15^ Self-efficacy connotes an individual’s confidence regarding the ability to engage in a particular behaviour,^16^ it predicts health behaviours, and further act as impetus to initiate and maintain physical activity and exercise during pregnancy.^17^ Thus, the theory of self-efficacy helps to explain the reasons for physical activity behaviour change, in this context, during pregnancy.^18^

Environmental, physical and cultural factors could shape the knowledge and attitudes of pregnant women regarding prenatal physical activity. Examining the knowledge and attitudes of pregnant women towards prenatal physical activity or exercise, a topic rarely studied in the context of South Africa, would inform possible intervention strategies. Such intervention strategies could improve women’s knowledge of prenatal physical activity; it could possibly address women’s negative attitudes and craft measures to sustain their positive attitude or behavior concerning prenatal physical activity. Prenatal physical activity or exercise is, and should be, part of the antenatal healthcare agenda in both primary and clinical settings. In this context, this present study assesses the knowledge and attitudes of pregnant women towards physical activity and exercise during pregnancy in the Eastern Cape, South Africa.

## METHODS

This cross-sectional study of 1082 convenient sample of pregnant women was conducted in 12 randomly selected antenatal primary health clinics in Buffalo City Municipality, Eastern Cape province, South Africa. We included pregnant woman over 18 years, receiving antenatal healthcare at the primary healthcare clinics, ability to read and speak the IsiXhosa, Afrikaans or English languages, and with singleton pregnancy. We excluded women with disabilities or reasons to stop exercise at the time of the study, based on the American College of Obstetricians and Gynaecologists (ACOG) recommendations,^19^ as previously reported in our earlier study.^20^

### Measures:

The demographic questionnaire solicited information on the age, residence, ethnicity, marital status, level of education, employment status, religion, and whether they received family support from family. Furthermore, the behavioural and lifestyle characteristics was determined by asking the participants their current exposure to alcohol during pregnancy and smoking. We also asked the participants whether they had had antepartum haemorrhage in their first trimester, and how they perceived their general health. In addition, the women were asked whether they received physical activity advice from health providers during pregnancy, and had participated in physical activity before and during pregnancy. However, information on parity, pregnancy delivery mode, and pre-pregnancy body mass index were obtained from the antenatal medical records of the participants.

A structured knowledge and attitude questionnaire with pre-coded questions derivable from literature^21^,^22^ was used for data collection. We asked the women whether they had knowledge regarding the benefits, contraindications, and types of exercise during pregnancy. There were four general statements on the knowledge of physical activity and 10 items defining the contraindications of physical activity in pregnancy. The participants were required to provide a “*yes”*, “*no”* or “*don’t know”* response to each of the statement. Likewise, we solicited participant’s knowledge regarding the types of physical activity during pregnancy. The questionnaire on attitudes towards prenatal physical activity had 13 items on a 5-point Likert scale ranging from 1 (strongly agree) to 5 (strongly disagree).

### Ethics approval:

The study protocol received ethical approval from the University of Fort Hare’s Human Research Ethics Committee (Ref#2019=06=009=OkaforUB). The study was conducted according to “Declaration of Helsinki” ethical research principles,

### Data collection:

Both demographic and the knowledge and attitude questionnaires were self-administered to pregnant women at the selected health clinics between July to October 2019.

### Data analysis:

The descriptive statistics of mean, frequency and percentages was applied. The participants responses on women’s knowledge and attitude toward physical activity during pregnancy were analysed as “strongly agreed” and “agreed” as one variable; likewise, the responses on “disagree” and “strongly disagreed” as a single variable. Each response was assigned a numerical value; and a high score indicated a more positive response. All statistical analyses were carried out using the Statistical Package for Social Sciences (SPSS) (Version 24.0, IBM SPSS, Chicago, IL, USA).

### Operational definitions:

Participants knowledge on physical exercise during pregnancy was classified as *high* or *low*. High knowledge about physical activity entails whether a participant knew and could answer correctly a statement pertaining to the types, appropriate or relevant antenatal exercises, and contraindications of exercises during pregnancy. Conversely, low knowledge means a participant is not aware and could not provide a correct statement on the types, benefits, and the contraindications of exercise during pregnancy. In addition, the attitude of women relating to prenatal physical activity practice was scored as correct answer = 1 score, incorrect (No) = minus score, unsure= 0 score. Therefore, participants with plus score were considered positive, while 0 or minus score was considered negative.

## RESULTS

The participants’ mean age was 27.0 years (Standard deviation = 6.2 years). The participants were mostly between the age of 19-34 years (*n* = 812; 75.1%) and living in urban settings (*n* = 523; 48.3%). They were mostly blacks (*n* = 935; 86.4%), never married (*n* = 717; 66.3%), and with a secondary education (*n* = 803; 74.2%). A majority of the participants were unemployed (*n* = 733; 67.7%) and had the support of their families (*n* = 837; 77.4%). The majority of participants were nulliparous (*n* = 517, 47.8%), had no antepartum haemorrhage (*n* = 1013; 93.6%), and had both vaginal and Caesarian (*n* = 538; 49.7%) delivery. An overwhelming majority of the participants had normal pregravid body mass index (18.5-24.9 kg/m^2^) (*n* = 909; 84.8%) and 753 (69.6%) had never participated in physical activity during pregnancy (data not shown).

### Knowledge on prenatal physical activity:

The knowledge of participants on the contraindications of prenatal physical activity (Table-I) indicated that the vast majority of participants had no knowledge of antennal exercise (65.8%), and indicated that physical activity during pregnancy is safe (88.2%) and beneficial for the baby (79.6%). In addition, the majority of the participants exhibited good knowledge by disagreeing with the statement that exercise during pregnancy would result in swelling of lower extremities (56.1%), extreme weight gain or loss (78.7%) and back pain (46.7%). Conversely, the overwhelming majority of the participants affirmed that exercise during pregnancy would result in difficulty in breathing (78.9%), chest pain (84.9%), dizziness (94.8%), uterine contractions (82.9%), incompetent cervix and preterm labour (82.3%), and vaginal bleeding (82.5%).

**Table-I:** Participants knowledge on benefits and contraindications of exercise during pregnancy.

Knowledge aspect	Yes *n* (%)	Don’t Know *n* (%)	No *n* (%)
Are you aware of antenatal exercise?	346 (32.0)	24 (2.2)	712 (65.8)
Physical activity in pregnancy is beneficial for the baby	861 (79.6)	139 (12.8)	82 (7.6)
Physical activity in pregnancy is safe	954 (88.2)	102 (9.4)	26 (2.4)
Healthy eating and physical activity are good	1051 (97.1)	25 (2.3)	6 (0.6)
***Contraindications***			
Swelling of lower extremities	290 (26.8)	185 (17.1)	607 (56.1)
Extreme weight gain or loss	118 (10.9)	113 (10.4)	851 (78.7)
Back pain	439 (40.6)	138 (12.7)	505 (46.7)
Difficulty in breathing	854 (78.9)	83 (7.7)	145 (13.4)
Chest pain	919 (84.9)	55 (5.1)	108 (10.0)
Dizziness	1026 (94.8)	29 (2.7)	27 (2.5)
Uterine contractions	897 (82.9)	90 (8.3)	95 (8.8)
Incompetent cervix and preterm labour	890 (82.3)	92 (8.5)	100 (9.2)
Vaginal bleeding	892 (82.5)	116 (10.7)	74 (6.8)
Diabetes	337 (31.1)	202 (18.7)	543 (50.2)

**Fig.1:**
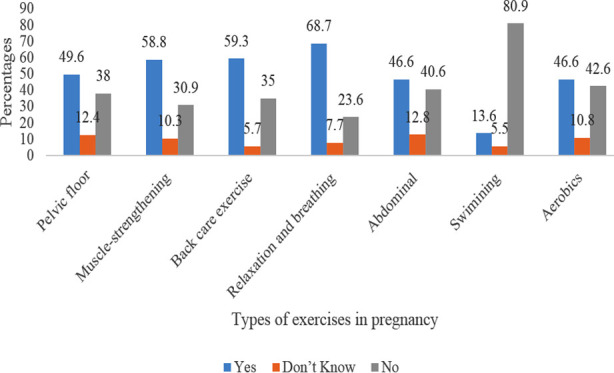
Participants knowledge regarding different types of exercises during pregnancy.

The participants had knowledge on pelvic floor exercise (49.6%), muscle-strengthening exercise (58.8%), back care exercise (59.3%), relaxation and breathing exercise (68.7%), abdominal exercise (46.6%), and aerobics exercise (46.6%). Conversely, majority of them had no knowledge of swimming exercise (80.9%) as part of antenatal exercise during pregnancy (Fig.1).

### Attitude towards prenatal physical activity:

Most of the participants expressed positive attitudes toward physical activity and exercise during pregnancy (Table-II). The participants expressed that healthy physical activity during pregnancy is an enjoyable experience (50.9%) and it prepared them for a healthy birth (73.3%). They refuted the statements that engaging in healthy physical activity is a waste of time (77.0%); makes them feel guilty (58.6%), and harms the baby (67.1%). The participants also affirmed that regardless of the busy schedule (49.9%) and care of children (64.6%), they are not afraid of physical activity/exercise (68.0%) during pregnancy. On the other hand, negative attitudes towards physical activity/exercise expressed by the participants included the feeling of tiredness (67.7%), lack of interest in physical activity/exercise (64.8%), inadequate information on physical activity/exercise (59.5%), and difficulty in achieving healthy physical activity/exercise during pregnancy (59.7%).

**Table-II:** Attitudes towards physical activity and exercise.

Attitude	Response distribution				

	SA n (%)	A n (%)	N n (%)	D n (%)	SD n (%)
I enjoy engaging in healthy physical activity during pregnancy	11(10.3)	439(40.6)	120(11.1)	261(24.1)	150(13.9)
Healthy physical activity makes me fit for birth	135(12.5)	658(60.8)	115(10.6)	94 (8.7)	80 (7.4)
Healthy physical activity in pregnancy is waste of time	10 (0.9)	155(14.3)	84 (7.8)	612(56.6)	221 20.4)
I feel guilty by not doing physical activity during pregnancy	61 (5.6)	327(30.2)	61 (5.6)	439(40.6)	194(18.0)
Physical activity harms the health of the baby	40 (3.7)	242(22.4)	74 (6.8)	544(50.3)	182(16.8)
I feel tired to exercise	146(13.5)	587(54.2)	26 (2.4)	216(20.0)	107 (9.9)
I do not feel like exercising	108(10.0)	593(54.8)	16 (1.5)	257(23.7)	108(10.0)
I have busy schedule	113(10.4)	413(38.2)	27(2.5)	307(28.4)	222(20.5)
I have children to care for	91 (8.4)	261(24.1)	31 (2.9)	445(41.1)	254(23.5)
I am afraid of exercise	57 (5.3)	249(23.0)	4(3.7)	521(48.1)	215(19.9)
I do not have sufficient information on exercise	126(11.6)	518(47.9)	13(1.2)	303(28.0)	122(11.3)
I dislike physical activity during pregnancy	93 (8.6)	495(45.8)	16(1.5)	350(32.3)	128(11.8)
It is difficult to achieve healthy physical activity	84 (7.8)	562(51.9)	29(2.7)	289(26.7)	118(10.9)

***Note:*** SA: strongly agree; A: agree; N: neutral; D: disagree; SD: strongly disagree.

**Fig.2:**
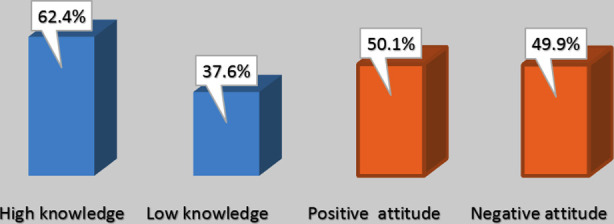
Frequency distribution of knowledge and attitude levels.

Overall knowledge and attitudes regarding physical activity among pregnant mothers (Fig.2) showed that the majority (62.4%) had high knowledge regarding prenatal physical activity. Similarly, half of the women showed a positive attitude toward prenatal physical activity (50.1%).

## DISCUSSION

To our knowledge, this is the first study exploring knowledge and attitudes towards physical activity and exercise among pregnant women in the Eastern Cape Province in South Africa. Exploring the factors that influence the decision of pregnant women to initiate and participate in physical activity or exercise during pregnancy have the potential of identifying the areas of weakness, and based on this, may help to design interventional strategies to improve maternal healthcare of women in the context of physical activity health outcomes. Overall, the finding of this current study revealed that the majority of the women (62.4%) had a high knowledge regarding physical activity. The women were aware of the safety and benefits of physical activity for the mother and baby, all of which have some support in the literature.^1^-^5^ Notably, the ACOG^5^ has categorically stated “in the absence of obstetric or medical complications or contraindications, physical activity in pregnancy is safe and desirable, and pregnant women should be encouraged to continue or to initiate safe physical activities”. Our study’s findings support those of studies conducted among pregnant women in Kenya,^23^ Nigeria,^24^ and Saudi Arabia,^25^ which shows women’s awareness regarding the benefits accruable from participation in prenatal physical activity. However, knowledge does not automatically translate into practice. Other studies have reported that, although women recognise the importance of prenatal physical activity, they rarely translate the knowledge into practice by participating in physical activity.^26^,^27^ Therefore, efforts to improve women’s knowledge is advocated as this would affect change in their attitudes and practices regarding prenatal physical activity.

Our findings show that women have mixed knowledge about the contraindications of exercise during pregnancy. While others refute that exercise does not result in swelling of lower extremities, extreme weight gain or loss, and back pain, the majority of the women hold an opposite view that exercising during pregnancy would result in difficulty in breathing, chest pain, dizziness, uterine contractions, incompetent cervix and preterm labour, and vaginal bleeding. In the study of Mbada et al.^21^ the majority of the Nigerian women under study indicated that exercise in pregnancy results in lesser risk of back pain (75.9%), prevention of excess weight gain (69.1%), and increased ability to cope with labour and delivery (69.6%).

Consistent with other studies, women in this present study had knowledge on the types of antenatal exercises, such as muscle-strengthening, back care, relaxation and breathing, abdominal, and aerobics exercises. Our finding confirmed similar findings about pregnant women studied in Nigeria,^21^ and Pakistan.^28^

Concerning the types of antenatal exercises and consistent with other studies,^21^,^22^ the majority of the participants had no knowledge of swimming exercise (80.9%) as part of antenatal exercise during pregnancy. However, walking, stationary cycling, aerobic exercises, dancing, resistance exercises (for example, using weights, elastic bands), stretching exercises, hydrotherapy, and water aerobics are, reportedly, safe exercises to engage in during pregnancy.^5^ The women in this present study are mostly from low-socio economic backgrounds, and may lack the resources to own or construct a swimming pool, possibly lack the skills to swim, and on another caveat, their knowledge of swimming may be limited and shaped by cultural beliefs and the concept of hydrophobia.^21^ However, these variables were not assessed; therefore, one cannot ascertain their influence on the lack of knowledge on swimming as an antenatal exercise.

The present study showed half of the women having expressed positive attitudes towards physical activity during pregnancy. This is similar to previous studies conducted among Nigerian,^21^,^24^ Pakistani,^28^ Australian,^29^ Indonesian,^30^ and Sri Lankan^31^ pregnant women, which reported positive attitudes toward antenatal exercise. Nowadays, the negative perception regarding the safety of prenatal physical activity has relatively improved, based on scientific evidence demonstrating manifold advantages of prenatal physical activity. Clearly, there is no evidence linking prenatal physical activity with maternal adverse outcomes,^1^-^4^ which hitherto seemed to be safety issues. Contrastingly, some pregnant women in our present study indicated negative (49.9%) attitudes towards physical activity, which was linked to tiredness, lack of interest, and inadequate information on physical activity. Our study echoes similar findings affecting the attitude of women to engage in prenatal physical activity.^22^,^26^,^28^,^32^ These modifiable barriers expressed by the women in our study highlight the need to tailor context-specific interventions to address their concerns. Attitude regulates one’s predisposition toward behaviour,^33^ and in this case, toward prenatal physical activity; therefore, information on women’s positive attitudes and knowledge on prenatal physical activity suggest interventions should target educational messages on the benefits and importance of engaging in prenatal physical activity to improve maternal health outcomes.

### Limitations of the study:

We included only pregnant women attending public primary health clinics in Buffalo City Municipality in the Eastern Cape Province, South Africa; therefore, the findings of the study cannot be generalized to other pregnant women attending private health facilities nor to the entire group of women in the province or South Africa. Notably, the definition of low knowledge in this present study is subject to varying perspectives of an individual, depending also on one’s level of accessibility to their various means, communication and experience. Notwithstanding these limitations, using a large sample of pregnant women, our study provides a unique insight into the efficacy and positive outcome of prenatal physical activity among women in an understudied, poorly resourced region.

## CONCLUSION

Most pregnant women are generally knowledgeable about prenatal physical activity; and while half of them expressed positive attitudes towards physical activity during pregnancy; they, however, identify lack of time, tiredness, lack of interest, and inadequate information, which are modifiable barriers, as being negative attitudes toward participation in physical activity. This finding points to the need to prioritize efforts in providing prenatal physical activity counselling as one of the important components of antenatal healthcare in the primary healthcare programme.

### Author`s Contribution:

**UBO:** Conceptualized the study, collected data and wrote the manuscript.

**DTG:** Read, interpreted the data, and critically reviewed the manuscript.

All authors contributed to the writing of the manuscript, read and approved the final version of the manuscript.
